# Comparison of DNA Methylation in Schwann Cells before and after Peripheral Nerve Injury in Rats

**DOI:** 10.1155/2017/5393268

**Published:** 2017-03-26

**Authors:** Xian-Hu Zhou, Wei Lin, Yi-Ming Ren, Shen Liu, Bao-You Fan, Zhi-Jian Wei, Gui-Dong Shi, Xin Cheng, Yan Hao, Shi-Qing Feng

**Affiliations:** ^1^Department of Orthopedics, Tianjin Medical University General Hospital, Tianjin, China; ^2^Tianjin Neurological Institute, Key Laboratory of Post-Neuroinjury Neuro-Repair and Regeneration in Central Nervous System, Ministry of Education and Tianjin City, Tianjin, China

## Abstract

This study aims to find the difference of genomewide DNA methylation in Schwann cells (SCs) before and after peripheral nerve system (PNS) injury by Methylated DNA Immunoprecipitation Sequencing (MeDIP-Seq) and seek meaningful differentially methylated genes related to repairment of injured PNS. SCs harvested from sciatic nerve were named as activated Schwann cells (ASCs), and the ones harvested from brachial plexus were named as normal Schwann cells (NSCs). Genomic DNA of ASCs and NSCs were isolated and MeDIP-Seq was conducted. Differentially methylated genes and regions were discovered and analyzed by bioinformatic methods. MeDIP-Seq analysis showed methylation differences were identified between ASCs and NSCs. The distribution of differentially methylated regions (DMRs) peaks in different components of genome was mainly located in distal intergenic regions. GO and KEGG analysis of these methylated genes were also conducted. The expression patterns of hypermethylated genes (Dgcr8, Zeb2, Dixdc1, Sox2, and Shh) and hypomethylated genes (Gpr126, Birc2) detected by qRT-PCR were opposite to the MeDIP analysis data with significance (*p* < 0.05), which proved MeDIP analysis data were real and believable. Our data serve as a basis for understanding the injury-induced epigenetic changes in SCs and the foundation for further studies on repair of PNS injury.

## 1. Introduction

Schwann cells (SCs) play an important role in the peripheral nervous system (PNS) and have been shown to have a variety of functions especially in participating in the formation of myelin, maintaining the morphology and function of myelin sheath as well as involving in the regeneration of nerve injury [1–4]. Particularly when PNS was injured, biological functions of SCs changed significantly and cannot be replaced. In response to nerve injury, a series of complex changes will occur at the distal end of the nerve, and this change process is called Wallerian degeneration [5]. With axonal degeneration, SCs are activated and proliferating rapidly, which helps to clear myelin and axon debris, guide axonal regrowth, and provide neurotrophic support [6–9]. Besides, SCs dedifferentiate into repair cells that are essential for axonal regeneration and then differentiate into myelinating SCs to restore nerve function [10].

Recently, a large number of studies have been focusing on epigenetics research of SCs related to PNS injury, and some studies [11–14] have showed that microRNAs and histone deacetylases of SCs emphasized the importance of epigenetic mechanisms for peripheral myelination after PNS injury. What is more, some studies [10] have reported that myelination of SCs can be regulated by DNA methylation process. Although previous studies [15, 16] have provided some clues for understanding the relationship between DNA methylation and gene regulation of SCs, due to the limitation of experimental technique, the mechanisms driving all of this still are not understood very well. In this study, for the first time, we comprehensively characterized the differences of genomewide DNA methylation expression regarding SCs before and after PNS injury by Methylated DNA Immunoprecipitation Sequencing (MeDIP-Seq). This research aims to seek meaningful differentially methylated genes, allowing deeper insight into molecule mechanisms of SCs during PNS injury process.

## 2. Methods and Materials

### 2.1. Animals and Surgery

Adult Wistar rats (*n* = 6, 180–200 g) were obtained from the Animal Center of Tianjin Medical University. Sciatic nerve injury surgeries were done as described previously [17]. Rats were anesthetized by intraperitoneal injection of ketamine (75 mg/kg). Under aseptic conditions, muscles and skins were carefully sutured. Bilateral sciatic nerves were exposed and ligated with silk thread on the proximal end of the sciatic nerve. All the experimental procedures involving animals were conducted according to Institutional Animal Care guidelines of Tianjin Medical University, China, and approved ethically by the Ethics Committee of Tianjin Medical University, China.

### 2.2. Schwann Cells Isolation and Culture

After 7 days, sciatic nerves and brachial plexus were acquired from 6 Wistar rats. After washing away the blood in phosphate-buffered saline (PBS) and removing the epineurium and connective tissue under the dissecting microscope, the tissues were cut into 1 × 1 mm explants with scissors [18]. SCs harvested from sciatic nerve were named as activated Schwann cells (ASCs), and the ones harvested from brachial plexus were named as normal Schwann cells (NSCs). Respectively, these tissues were digested in 5 mg/mL type I collagenase at 37°C for 7 min and in 0.25% trypsin (Sigma # T4549) for 5 min. The density of SCs was adjusted to 1 × 10^5^/mL. SCs were cultured in Dulbecco's modified Eagle's medium (DMEM) with 50 ug/mL penicillin-streptomycin on polylysine-coated dishes at 37°C and 5% CO_2_. When SCs covered 85% of bottom of flask, the passage cells were digested with 0.25% trypsin (37°C, 5 min).

### 2.3. Immunohistochemistry

The purified SCs were cultured to the third generation and were planted on poly-D-lysine-coated coverslips in 24-well plates. After 72 hours, SCs were briefly washed with PBS and fixed with 4% paraformaldehyde for 15 minutes. Then these cells were washed 3 times with PBS and permeabilized with 0.5% Triton X-100 15 min. After blocking with 0.5% (v/v) goat serum in PBS for 1 hour at room temperature, these cells were incubated with S100ß antibody (mouse anti-rat antibody, 1 : 500 volume dilution) overnight at 4°C. After washing 3 times in PBS, SCs were incubated with conjugated secondary antibodies (FITC) goat anti-mouse IgG for 1 hour (diluted 1 : 1000 in PBS). Lastly, the nuclei were stained with DAPI and images were captured using a laser scanning confocal microscope.

### 2.4. Cell Proliferation Assay

Cell Counting Kit-8 (CCK-8, Dojindo, Japan) was used in cell proliferation assays. These cells were seeded into 96-well plates, and 3 duplicate wells were set. At the same time 3 control wells were set correspondingly. The density of cells was 1000 per well with 100 uL growth medium. Then cells were treated with the CCK8 (10 uL, 2 hours per well) and measuring the numbers of cells per well was conducted by the absorbance (450 nm) of reduced water-soluble tetrazolium salt (WST) for 7 days continuously.

### 2.5. High-Throughput MeDIP Sequencing Analysis

**Figure 1:**
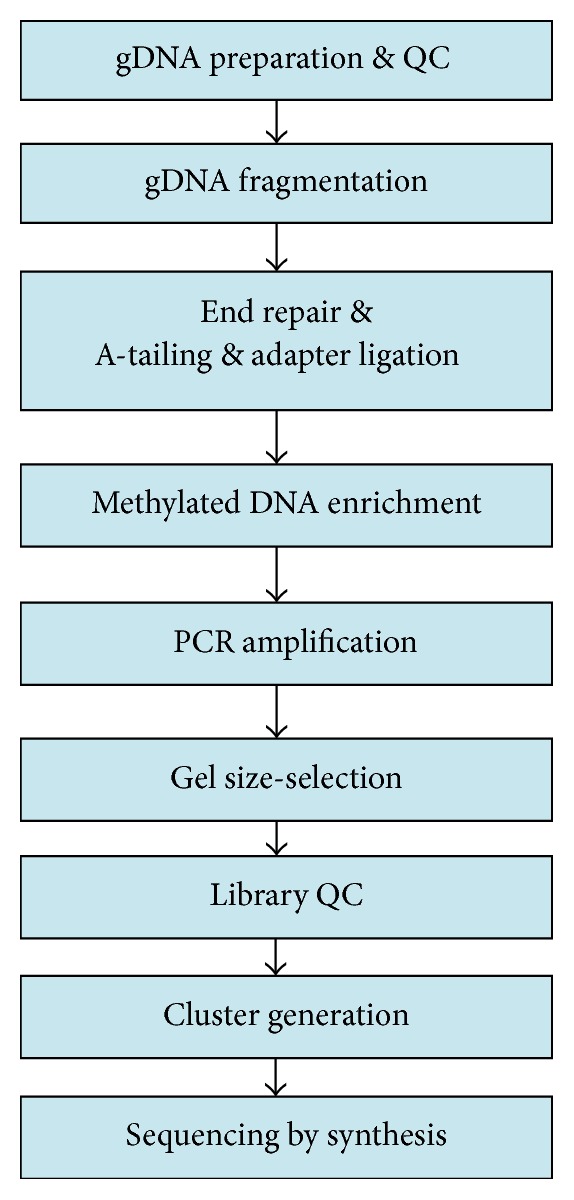
Concise experimental procedure for DNA methylation sequencing.

The genomic DNA of ASCs and NSCs was extracted and purified, respectively. Then sonication method was used to fragment these DNA into 100–500 bp fragments. DNA fragment 3′ end repair was carried out by adding Adenine (A) and sequencing adapters were simultaneously added to their ends by Paired-End DNA Sample Prep Kit (Illumina, USA). Double stranded DNA was denatured as single stranded DNA. Subsequently, sequencing adapters-ligated DNA fragments and 5-methylcytosine (5-mC) antibody beads (Diagenode, USA) were immunoprecipitated. These methylated DNA fragments were enriched. Then immunoprecipitated products were amplified and validated by quantitative real-time polymerase chain reaction (qRT-PCR), and those of 200–300 bp were excised from the gel and purified. DNA library quality control and sequencing run were processed by Illumina HiSeq 2000 platform (Illumina, USA). Finally, we got the Illumina sequencing raw data of DNA. Concise experimental procedure for DNA methylation sequencing was shown in Figure 1.

### 2.6. Data Analysis

Data analysis was carried out according to the following steps. First, the previous sequencing data of DNA was used and the high quality data of them were screened out according to the screening criteria as follows: (1) Nitrogenous (N) base content of each read is not more than 5%; (2) low quality base is not more than 30%; (3) read does not contain an adapter sequence. Second, the high quality read was mapped to the latest rat genome assembly (http://hgdownload.soe.ucsc.edu/goldenPath/rn5/bigZips/). Read distribution analysis containing the distribution in rat chromosomes and differentially methylated regions (DMRs) (such as promoter (≤1 kb), promoter (1-2 kb), 1st exon, other exons, 1st intron, other introns, 3′ UTR, 5′ UTR, distal intergenic regions, and downstream (≤3 kb) and CpG islands (CGIs)) was discovered from uniquely mapped reads. CpG islands were downloaded from UCSC database. Third, differentially methylated genes were screened according to *p* < 0.05 and log2(ASCs/NSCs) > 0 or log2(ASCs/NSCs) < 0. Last, by using the Model-based Analysis of ChIPSeq (MACS) V 1.4.2 (http://liulab.dfci.harvard.edu/MACS/), the genomewide methylation peak scanning was performed and we analyzed the number of peaks in different components of the rat genome. Besides, the number of methylated peaks in the whole genome known as the total peak number and a peak overlapping different components were also analyzed. Gene ontology (GO) enrichment analysis and Kyoto Encyclopedia of Genes and Genomes (KEGG) pathway analysis were performed using DAVID6.7 database (http://david.abcc.ncifcrf.gov/).

### 2.7. qRT-PCR Verification

Seven genes were verified by qRT-PCR with the Bio-Rad CFX96 Real-Time PCR System (Bio-Rad, USA). Total RNA was isolated from the ASCs and NSCs by using TRIZOL (Invitrogen Corp, Carlsbad, CA) and was polyadenylated and reverse-transcribed with a poly(T) adapter into cDNA following the manufacturer's directions. Real-time PCR was performed using SYBR green dye in a thermal cycler with the following parameters: an initial denaturation step at 95°C for 30 min; 40 cycles at 95°C for 5 seconds and 60°C for 30 seconds. Complete experimental process was performed for each sample in triplicate. All primers were synthesized by Shanghai Shenggong Inc, and mRNA-specific primers were listed in Table S2, in Supplementary Material available online at https://doi.org/10.1155/2017/5393268. All data were analyzed using the 2^−ΔΔCT^ method to calculate the difference between the threshold cycle (CT) values of the target genes in each sample.

### 2.8. Statistical Analysis

All data were analyzed using SPSS statistical software (version 11.5 for Windows). Statistical analysis was performed using two-tailed Student's *t*-test, and differences were considered statistically significant at *p* < 0.05.

## 3. Results

### 3.1. Identification of Schwann Cells

**Figure 2:**
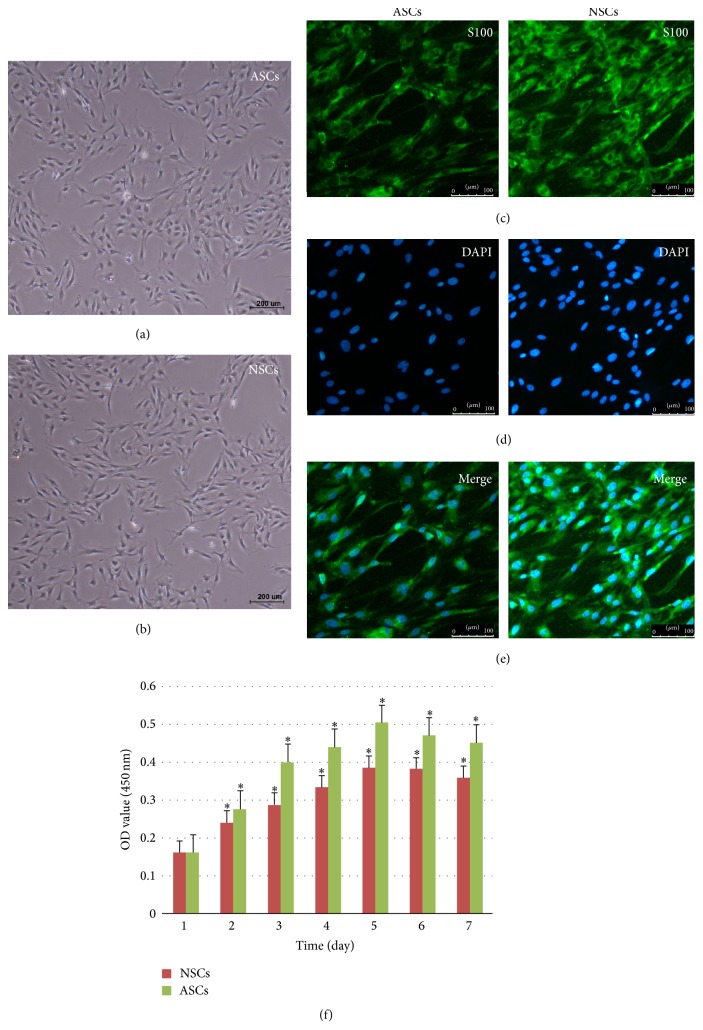
(a) The shape of activated Schwann cells (ASCs) under optical microscope. (b) The shape of normal Schwann cells (NSCs) under optical microscope. Both of these SCs, long spindle cells, were arranged in fish shape and nucleus was ovoid or oblong. Scale bar: 200 um. (c) SCs were marked with S-100ß by immunofluorescence. (d) The nucleus of SCs was marked with DAPI by immunofluorescence. (e) SCs and nucleus of SCs were merged together by immunofluorescence. Both of the two kinds of SCs had positive results under fluorescence microscope. Scale bar: 100 um. (f) The growth rate of SCs was showed by optical density (OD) value of 450 nm from 1 day to 7 days through cells proliferation assay. The growth rate of ASCs was obviously higher than that of NSCs starting from 2 days.

The results of observation under optical microscope showed no morphological differences between ASCs and NSCs (Figures 2(a) and 2(b)). SCs, long spindle cells, all were arranged in fish shape and nucleus was ovoid or oblong. Both of two kinds of SCs had positive results under fluorescence microscope after S100ß immunocytochemistry staining (Figures 2(c)–2(e)). In the same culture condition, ASCs were faster to attach the wall than NSCs, and division rate and growth rate of ASCs were higher than those of NSCs (Figure 2(f)).

### 3.2. Methylomic Profiling of ASCs and NSCs

After MeDIP-Seq, we generated a total of 6,592,337,700 bp data from ASCs groups and 7,136,742,100 bp data from NSCs groups. 17474976 reads were obtained in NSCs groups and 16816796 reads in ASCs groups were obtained, respectively. To our satisfaction, 16415908 reads of NSCs group and 15729752 reads of ASCs group were uniquely mapped to the latest rat genome, which showed that more than 93% of MeDIP-Seq reads were aligned (mapped) on latest rat genome in each group. In addition, more than 80% of MeDIP-Seq reads were high quality (HQ) mapped reads in Table 1. Besides, sequencing statistics revealed that all six samples performed well, with anticipated GC content skewedness of all mapped reads (Supplementary Material 1: Table S1).

### 3.3. Characterization of Differential Methylated Regions

**Figure 3:**
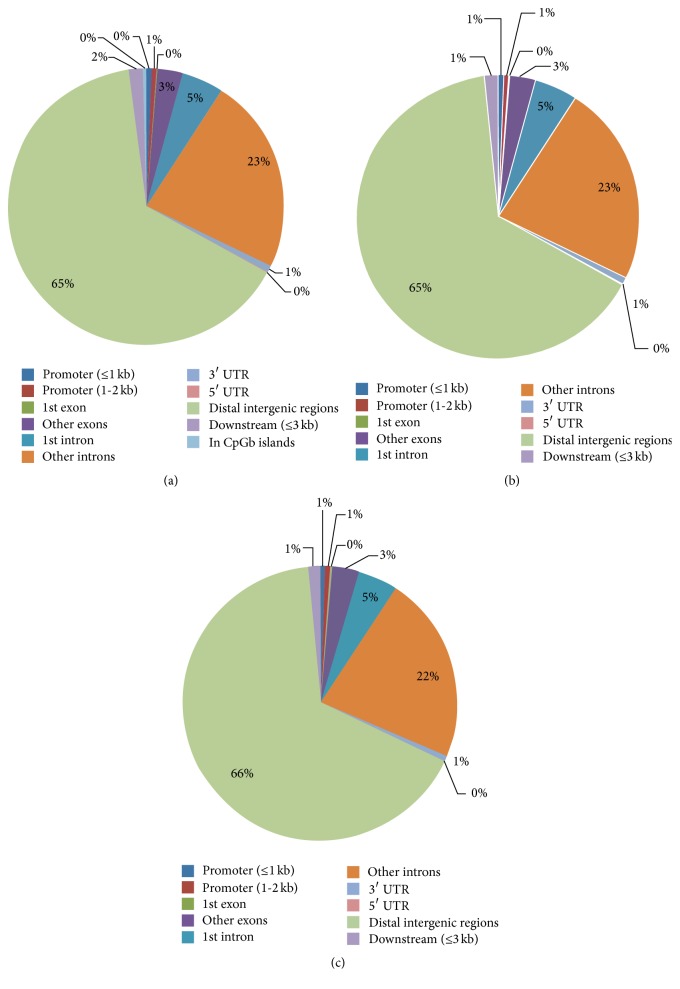
(a) The distribution of differentially methylated regions (DMRs) peaks in different components of genome. (b) The distribution of DMRs peaks in activated Schwann cells (ASCs). (c) The distribution of DMRs peaks in normal Schwann cells (NSCs).

A total of 13005381 HQ mapped reads in ASCs group and 13137926 HQ mapped reads in NSCs group were distributed in the chromosomes of the DMRs. Of them, HQ mapped reads mainly were found in chr1 and detailed results were showed in Table 2. There are 11610434 genome regions in total and 176,610 DMRs were identified. Among DMRs, 44.1% were hypermethylated and 55.9% were hypomethylated in Table 3. The DMRs were all located in promoter (≤1 kb), promoter (1-2 kb), 1st exon, other exons, 1st intron, other introns, 3′ UTR, 5′ UTR, distal intergenic regions, downstream regions (≤3 kb), and CGls in Figure 3(a). The distribution of DMRs peaks in different components of genome showed that uniquely mapped reads in distal intergenic regions had a relatively higher methylation level than others (65.10% in NSCs group, 66.43% in ASCs group) in Figures 3(b) and 3(c). Only 567 DMRs peaks in CGls account for only 0.32 percent. Assessing the enrichment of methylated regions revealed that the relative frequency of CpGs within the regions and the observed/expected ratio of CpGs within the regions were above 1 in all cases between three ASCs groups and three NSCs groups, indicating a successful enrichment of methylated fragments in the data sets (Table 4).

### 3.4. Hypermethylated or Hypomethylated Genes

A total of 176612 methylated genes between ASCs and NSCs were detected by high-throughput MeDIP sequencing analysis (*p* < 0.05). Among them, 77800 genes were hypermethylated and 98812 genes were hypomethylated. According to screening criteria, fold change and whether CGls of these genes are methylated, only 567 methylated genes caught our attention. 433 hypermethylated genes were discovered such as Gpr4, Dgcr8, Zeb2, Dixdc1, Sox2, Shh, Lrfn3, Det1, Shox2, Abracl, Trdn, Irx2, Pabpc6, Sbk1, and Peg3. 134 hypomethylated genes which included Gpr126, Birc2, Sepw1, Irgq, Atp5sl, Hipk4, Ttc9b, Tulp2, Bex1, Mex3b, Ctsc, Gucy2d, Rrm1, Fam160a2, and Cdkn1c were revealed.

### 3.5. GO and KEGG Analysis of Methylated Genes as Functional Categories

**Figure 4:**
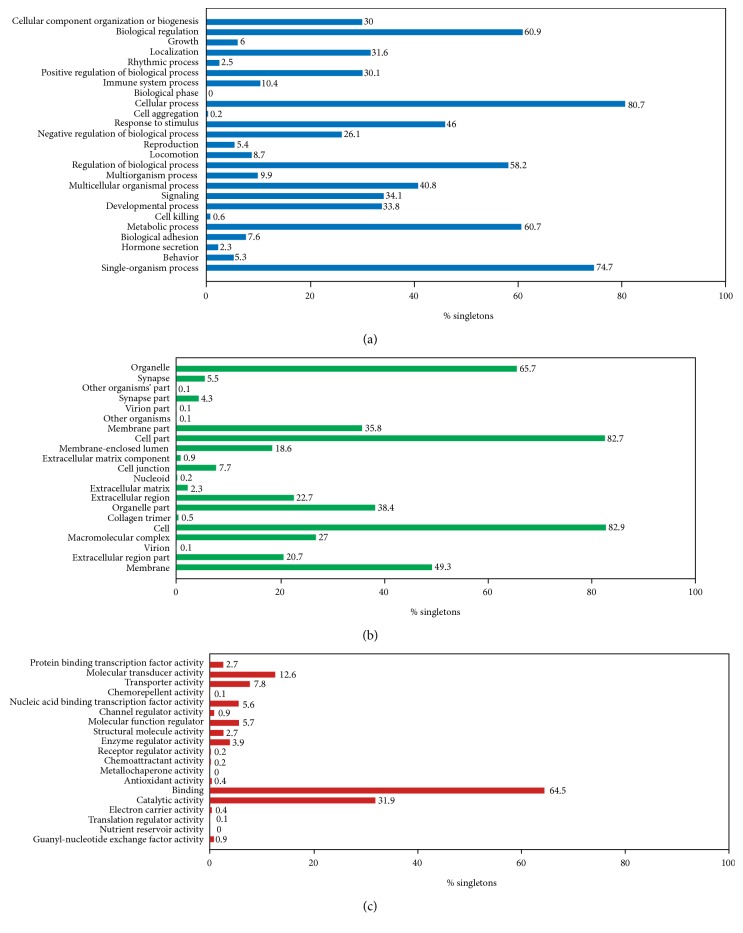
GO-enrichment analysis of biological processes (a), cellular components (b), and molecular functions (c).

Hypermethylated and hypomethylated genes were significantly enriched (*p* < 0.05) in GO-enrichment analysis of biological processes, molecular functions, and cellular components. Among biological process, methylated genes were mainly associated with biological regulation, cellular process, regulation of biological process, metabolic process, single-organism process, and others in Figure 4(a). Among molecular function, methylated genes were enriched into the category of binding activity, catalytic activity, molecular transducer activity, transporter activity, and others in Figure 4(b). In addition, methylated genes are predominantly involved in the cellular component including cell part, organelle, membrane, extracellular region, and others in Figure 4(c).

Signaling pathway analysis of hypermethylated and hypomethylated genes was conducted. A total of 279 signaling pathways were identified. Of them, 9 signaling pathways including cAMP signaling pathway, Wnt signaling pathway, ERK signaling pathway, PI3-Akt signaling pathway, Hippo/YAP signaling pathway, MAPK signaling pathway, Rap1 signaling pathway, cGMP-PKG signaling pathway, and calcium signaling pathway were considered to be significant (*p* < 0.05) in Table 5.

### 3.6. Genes Related to Repairment of Peripheral Nerve Injury

Differentially methylated genes related to repairment of peripheral nerve injury including adhesion, secretion, proliferation, neuronal regeneration, and axonal regeneration were uncovered. For adhesion, 580 methylated genes were identified, such as Sox-2, Shh, Lphn1, and Cdh9. 553 differentially methylated genes related to secretion including Lphn1, Pim3, and Plcd1 were found. We also confirmed 787 methylated genes associated with proliferation such as Dixdc1, Lrp6, Edn3, and Ephb1. For neuronal regeneration, 473 methylated genes were identified, like Bhlhb9, Cckar, Klhl1, Dlg2, and others. In addition, 215 differentially methylated genes related to axonal regeneration including Ifrd1, Unc5c, and Ntrk2 were found. Detailed results have been summarized in Table 6.

### 3.7. Genes Expression Validation by qRT-PCR

**Figure 5:**
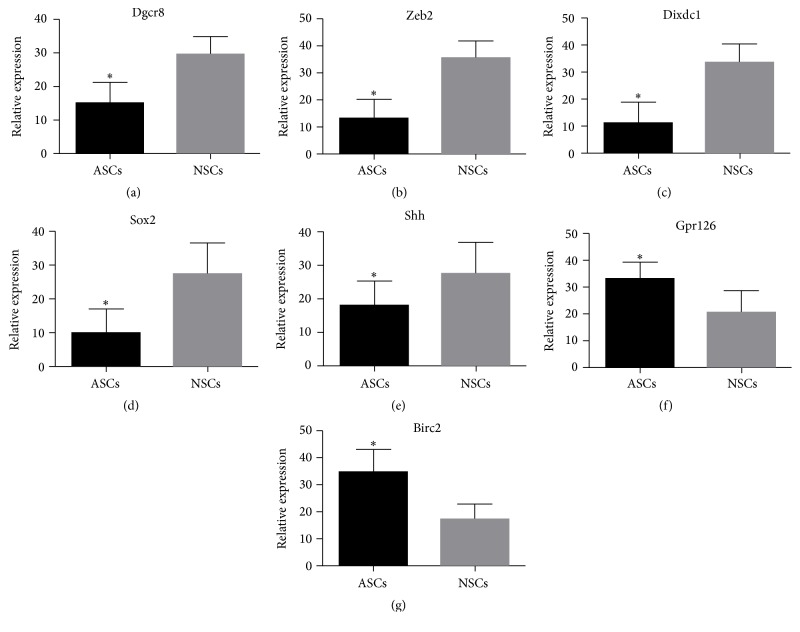
Validation of the differential expression of 7 mRNAs of corresponding methylated genes identified in the MeDIP in ASCs compared with NSCs by qRT-PCR. Data indicate relative expression following normalization. Values are means ± SE (^*∗*^*p* < 0.05). (a)–(e) showed that 5 mRNAs of differentially hypermethylated genes (Dgcr8, Zeb2, Dixdc1, Sox2, and Shh) were downregulated in ASCs compared with NSCs. (f)-(g) showed that 2 mRNAs of differentially hypomethylated genes (Gpr126, Birc2) were upregulated in ASCs compared with NSCs.

In addition to validating the MeDIP analysis results, qRT-PCR was used to quantify parts of mRNAs of corresponding methylated genes in ASCs compared with NSCs, such as 5 differentially hypermethylated genes (Dgcr8, Zeb2, Dixdc1, Sox2, and Shh) and 2 differentially hypomethylated genes (Gpr126, Birc2), resulting in the fact that they were closely related with repairment of peripheral nerve injury after searching PubMed. As shown in Figure 5, the mRNA expression patterns of Dgcr8, Zeb2, Dixdc1, Sox2, Shh, Gpr126, and Birc2 detected by qRT-PCR were opposite with the corresponding gene methylation alteration of MeDIP analysis data with significance (*p* < 0.05).

## 4. Discussion

Epigenetics is the branch of traditional genetics, whose meaning is that this action changes expression and function of genes and generates heritable phenotypes under the condition of unchanged DNA sequences [19]. DNA methylation is made up of DNA methyltransferase catalytic S adenosine armour sulfur chlorate as a methyl donor, so cytosine can be converted to 5-methyl cytosine [20]. Gene methylation can change configurations of gene, thus affecting transcription of transcription factor and gene expression. As an epigenetic silencing mechanism, DNA methylation plays essential roles in several developmental and cellular processes such as transcription, X-chromosome inactivation, and genomic imprinting [21, 22]. In this study, using high-throughput MeDIP-Seq, our data showed the different patterns of DNA methylation between ASCs and NSCs and provided a comprehensive, detailed picture of DNA methylation patterns and methylated gene expression levels in SCs, which helps to understand the DNA methylation mechanisms of SCs in repairing injured PNS.

ASCs serve a critical role in nerve remyelination after PNS injury, which are specialised glial cells and myelin-forming cells in PNS [23]. PNS injury leads to Wallerian degeneration in the distal stump and ASCs proliferate to form bands of Bungner as cellular scaffold, providing the essential guidance and support for regenerating axons [24, 25]. In addition, when PNS was injured, NSCs changed into ASCs to work. Our data proved once again that ASCs had better division rate and growth rate, which indicated that ASCs were more effective for structural and functional recovery of PNS injury.

DNA methylation peak number shows the popularity of methylation in genome. More DNA methylation peaks mean more loci in genome are methylated. Our data indicated that the distribution of DMRs peaks in different components of genome showed that uniquely mapped reads in distal intergenic regions had a relatively higher methylation level but only 567 DMRs peaks in CGls account for only 0.32 percent. It should also be noted that DNA methylation does not occur exclusively at CGls. In fact, recently tissue-specific DNA methylation has been found at CpG island shores, outlying areas close to CGls (up to 2 kb distance), and strongly related to gene expression inactivation [26, 27]. To sum up, the 567 DMRs peaks in CGls of our findings are considered to be meaningful in DNA methylation mechanisms of SCs in repairing injured PNS.

In order to disclose the underlying molecular mechanisms of SCs in repairing injured PNS, we characterized the possible GO functional terms and signaling pathways of differentially methylated genes using the GO function and KEGG analysis. Considering the results of GO function analysis, we linked the differentially methylated genes with biological regulation, cellular process, regulation of biological process, metabolic process, single-organism process, and other biological processes which are probably very important for the injured PNS repair process of SCs. As previous articles reported, our KEGG pathway analysis showed that cAMP signaling pathway, Wnt signaling pathway, ERK signaling pathway, PI3-Akt signaling pathway, Hippo/YAP signaling pathway, MAPK signaling pathway, and others were among the most relevant pathways for injured PNS repair process of SCs. Calcitonin gene-related peptides could activate the cAMP-PKA-ERK signaling cascade, which may play a direct role in initiating inflammatory processes in the PNS [28, 29]. Tao et al. [30] discovered that carboxymethylated chitosan can promote the proliferation of cultured SCs and synthesis of nerve growth factor by activating the Wnt/b-catenin signaling pathway. Napoli et al. [31] identified a central role for ERK signaling in SCs in orchestrating nerve repair and it was a powerful system for studying peripheral neuropathies. By activating p38 MAPK and PI3K-Akt signal cascades, Netrin-1 could enhance SCs migration [32]. Chang et al. [33] deemed that Alpiniae Oxyphyllae Fructus could increase PAs and MMP2/9 and activate MAPK mediated signaling to induce SCs migration and nerve regeneration. Besides, Melfi et al. [34] highlighted a new discovery that Hippo/YAP signaling pathway participated in forming tight junction and cell-to-cell adhesion in SCs during PNS repair process. All these signaling pathways may play important roles in molecular mechanism of PNS repair process of SCs.

Also of note is that there were numerous evidences for our differentially methylated genes, which have proven to play important roles during PNS injury repairment. Glenn and Talbot [35] found that Gpr126 was essential for SCs to initiate myelination and addressed the role of Gpr126 signaling in myelin maturation and maintenance. Dgcr8 is responsible for modulation of myelin formation and maintenance as well as suppression of an injury-related gene expression program in SCs [36]. Quintes et al. [37] proved that Zeb2 is essential for Schwann cell differentiation, myelination, and nerve repair. By upregulating CyclinD1 and downregulating p21, Dixdc1 could activate PI3K pathway to promote Schwann cell proliferation after sciatic nerve crush [38]. Wang et al. [39] deemed that Birc2 and Birc3 of SCs, which might be the most potential targets for antiapoptotic protection mediated by inflammatory cytokines, were mainly responsible for the inflammation-mediated antiapoptosis of peripheral nerves. Besides, c-Jun-modified SCs, which have the potential to promote axonal regeneration and functional recovery, could enhance proliferation and migration abilities compared with control cells [40]. Sox2, as a negative regulator of myelination, could inhibit Schwann cell differentiation and myelination [41]. In addition, Shh gene, which is not normally expressed in SCs, is activated upon nerve injury. Lin et al. suggested that Sox-2 and Shh were involved in adhesion in SCs [36]. The mRNA expression patterns of Dgcr8, Zeb2, Dixdc1, Sox2, Shh, Gpr126, and Birc2 detected by qRT-PCR were opposite to the MeDIP analysis data with significance (*p* < 0.05). CGls of these genes are hypermethylated or hypomethylated, which would inhibit or promote the expression of their corresponding mRNAs. So our qRT-PCR results proved that MeDIP analysis data were real and believable. Last but not least, the differentially methylated genes merely by qRT-PCR are not enough. Further experiments should be carried out to verify the CpG methylation of these differential methylated genes by bisulfite sequencing PCR (BSP).

## Supplementary Material

Table S1: Distribution of Reads GC in different Schwann cells groups.
Table S2: Primers of methylated genes validated by qRT-PCR.

## Figures and Tables

**Table 1:** Reads genome alignment statistics.

Class	ASCs		NSCs	
	Number	%	Number	%
Total reads	16816796		17474976	
Total mapped^a^ reads	15729752	93.54	16415908	93.94
Total HQ mapped reads	13005381	82.68	13137926	80.03
Promoter (≤1 kb)	90241	0.69	84170	0.64
Promoter (1-2 kb)	81955	0.63	79542	0.61
1st exon	20000	0.15	17909	0.14
Other exons	458938	3.53	430799	3.28
1st intron	625989	4.82	610668	4.65
Other introns	2953913	22.72	2887216	21.99
3′ UTR	91283	0.70	87248	0.66
5′ UTR	9569	0.07	9244	0.07
Distal intergenic regions	8463689	65.10	8722453	66.43
Downstream (≤3 kb)	204583	1.57	200206	1.52
In CpG^b^ islands	97569	0.62	79283	0.48

^a^Reads were mapped by BWA database with the default parameter.

^b^CpG islands were downloaded from UCSC database.

ASCs: activated Schwann cells; NSCs: normal Schwann cells; HQ: high quality.

**Table 2:** Chromosome distribution of total HQ mapped reads.

Class	ASCs		NSCs	
	Number	%	Number	%
Total HQ mapped reads	13005381		13137926	
chr1	1454481	11.1836862	1543628	11.7494
chr2	1074744	8.26384094	1098163	8.35872
chr3	816906	6.28129234	808555	6.15436
chr4	814638	6.2638534	810404	6.16843
chr5	754336	5.80018379	755245	5.74859
chr6	662551	5.09443745	661399	5.03427
chr7	742593	5.70989039	748669	5.69853
chr8	633675	4.87240628	627670	4.77754
chr9	604079	4.64483893	601617	4.57924
chr10	615858	4.73540914	597108	4.54492
chr11	406076	3.12236912	403700	3.07278
chr12	386267	2.97005524	381848	2.90646
chr13	645303	4.96181542	707230	5.38312
chr14	588247	4.52310471	584761	4.45094
chr15	503096	3.86836802	509972	3.88168
chr16	431447	3.31744991	434373	3.30625
chr17	488717	3.7578061	480979	3.661
chr18	431679	3.31923378	428225	3.25946
chr19	363485	2.7948816	353620	2.6916
chr20	372963	2.86775912	361241	2.7496
chrX	209019	1.60717322	231048	1.75863
chrM	5221	0.04014492	8471	0.06448

chr: chromosome; ASCs: activated Schwann cells; NSCs: normal Schwann cells; HQ: high quality.

**Table 3:** Differential methylation of Schwann cells statistics.

Class	Number	%
Genome regions	11610434	100
DMR	176610	1.52113177
Hypermethylated	77799	44.0512995
Hypomethylated	98811	55.9487005

DMR: differentially methylated region.

**Table 4:** Calculating CpG enrichment of provided short reads compared to the reference genome.

Class	CpGs number	CpGs rel. frequency (%)	CpGs obs/exp	Enrichment rel. frequency (%)	Enrichment obs/exp
BSgenome.Rnorvegicus.UCSC	24713205	0.851414595	0.247437644	—	—
ASCs_1	73092752	2.01814666	0.393297098	2.37034539	1.589479639
ASCs_2	66573452	1.986667228	0.38941539	2.333372296	1.57379202
ASCs_3	56781764	2.026584608	0.394905638	2.380255893	1.595980428
NSCs_1	68221902	1.916242372	0.381639103	2.25065718	1.542364761
NSCs_2	62275625	1.922886765	0.382886143	2.258461126	1.547404574
NSCs_3	67670669	1.982610575	0.391849804	2.328607692	1.583630515

rel: relative; obs: observed; exp: expected; ASCs: activated Schwann cells; NSCs: normal Schwann cells.

All statistics of calculations and plot are using MEDIPS, R package for MeDIP-Seq. BSgenome, Rnorvegicus, and UCSC were all genome databases.

**Table 5:** Significant KEGG signaling pathways of methylated genes.

Pathway	Gene number	*p* value	Partial related methylated genes
Calcium signaling pathway	136	0.01	Phka2, Adcy7, Hrh2, Drd1, Grm1, Vdac1, Grm5, Pde1b, Tnnc2, Nos1, Camk4
cAMP signaling pathway	143	0.02	Vav3, Drd2, Adcy10, Rhoa, Sox9, Mc2r, Gli3, Cngb3, Rras, Adcy1, Rac1
cGMP-PKG signaling pathway	126	0.02	Adcy7, Irs1, Npr2, Mylk, Rhoa, Adrb1, Ins2, Adcy5, Gna12, Itpr3, Prkg1
Wnt signaling pathway	105	0.03	Fzd5, Nlk, Smad4, Chd8, Plcb1, Sfrp4, Sost, Mapk8, Gsk3b, Lrp5, Rac1
Rap1 signaling pathway	150	0.03	Kit, Magi1, Krit1, Plce1, Pgf, Csf1, Tln1, Cdh1, Skap1, Fgf8, Rac1, Actg1
ERK signaling pathway	62	0.04	Mtor, Nck1, Erbb3, Egf, Araf, Pak7, Shc3, Plcg2, Ptk2, Tgfa, Nrg1, Kras
PI3-Akt signaling pathway	209	0.04	Itga4, Ccne1, Gng7, Cdc37, Tlr2, Ccnd3, Gngt1, Hgf, Syk, Fgf1, Tnr, Tnc
Hippo/YAP signaling pathway	104	0.04	Fzd5, Mpp5, Crb2, Gsk3b, Tead1, Ccnd1, Actb, Smad3, Dlg1, Fzd4, Bmp7
MAPK signaling pathway	172	0.04	Atf2, Cd14, Rras2, Ntf3, Rap1b, Grb2, Dusp1, Stk4, Mapk3, Jun, Nf1

KEGG: Kyoto Encyclopedia of Genes and Genomes.

**Table 6:** Five aspects of genes related to repairment of peripheral nerve injury.

Biological function	Gene number	Partial related methylated genes
Adhesion	580	Sox-2, Shh, Itpkb, Pkd1, Reln, Ptk2b, Nck2, Ass1 Lphn1, Cdh9, Epdr1, Myf5, Pdpn, Has2, Fgfrl1, Fer
Secretion	553	Lphn1, Pim3, Fst, Pim3, Pcsk6, Lax1, Sct, Sytl3 Plcd1, Ykt6, Jak1, Stat2, Myc, Csf2, Lifr, Sos1
Proliferation	787	Dixdc1, Lrp6, Edn3, Nkx2, Cyr61, Src, Sox8, Stk4 Ephb1, Sstr3, Rrm2, Tcf3, Grn, Rhoa, Apc, Nox4, Strn
Neuronal regeneration	473	Bhlhb9, Cckar, Fzd2, Thy1, Pbx3, Otx2, Lhx8, Btg2 Klhl1, Dlg2, Pak1, Wnt3, Mif, Tctn1, Evl, Ext1, Als2
Axonal regeneration	215	Ifrd1, c-Jun, Bcl2, Tnn, Mbp, Slit3, Ist1, Drgx, Thy1 Unc5c, Ntrk2, Isl1, Ptk2, Dscam, Atl1, Dnm2, Cxcl12
