# Supplementary material for: Comparison of DNA Methylation in Schwann Cells before and after Peripheral Nerve Injury in Rats
Source: Biomed Res Int. 2017 Mar 26;2017:5393268. doi: 10.1155/2017/5393268 (PMC5385226; doi:10.1155/2017/5393268)
Supplement: Supplementary file 1 — Table S1: Distribution of Reads GC in different Schwann cells groups. Table S2: Primers of methylated genes validated by qRT-PCR. [file 5393268.f1.pdf]

Table S1. Distribution of Reads GC in different Schwann cells groups

GC(%)	ASCs_1		ASCs_2		ASCs_3		NSCs_1	
	number	%	number	%	number	%	number	%
1-10	184	0.00	158	0.0013	135	0.0013	148	0.0011
11-20	10293	0.08	9570	0.0789	7283	0.0726	11179	0.0851
21-30	323152	2.48	307257	2.5322	238650	2.3802	357232	2.7191
31-40	3002145	23.08	2978224	24.5449	2338347	23.3217	3382174	25.7436
41-50	5857217	45.04	5382716	44.3614	4504813	44.9291	5852141	44.5439
51-60	3398380	26.13	3077875	25.3662	2628824	26.2188	3177854	24.1884
61-70	392842	3.0206	356171	2.9354	296520	2.9574	340067	2.5884
71-80	20041	0.1541	20375	0.1679	11259	0.1123	16099	0.1225
81-90	1127	0.0087	1436	0.0118	661	0.0066	1032	0.0079
91-100	5	0	1	0	2	0	2	0

ASCs Activated Schwann cells; NSCs normal Schwann cells; HQ high-quality; G Guanine; C Cytosine.

Table S2. Primers of methylated genes validated by qRT-PCR

Gene	Forward	Reverse
Dgcr8	5’-GCATGTGTTCCTTCTGCTCTGAT-3’	5’-CAGGACGCACTGAGGAGGTAGT-3’
Zeb2	5’-TAGCCGGTCCAGAAGAAATG-3’	5’-GGCCATCTCTTTCCTCCAGT-3’
Dixdc1	5’-ATCCATCAAGCACCATCAAC-3’	5’-CCCAAACTTTCCCAGTCGT-3’
Sox2	5’-TGGACAGTTACGCGCACAT-3’	5’-CGAGTAGGACATGCTGTAGGT-3’
Shh	5’-CTGCTGGTATGCTCGGGACTG-3’	5’-TAAATCGCTCGGAGTTTCTGG-3’
Gpr126	5’-CCAAAGTTGGCAATGAAGGT-3’	5’-GCTGGATCAGGTAGGAACCA-3’
Birc2	5’-CAGCTTTGTGCAGACTTTGCTTTC-3’	5’-CCTTGTTCCAGAGGTAGCGAGTG-3’
GAPDH	5’-AGAGCTACGAGCTGCCTGAC-3’	5’-AGCACTGTGTTGGCGTACAG-3’
